# MIDDLE AGED AND OLDER BLACK AMERICAN PERCEPTIONS OF DISCRIMINATION

**DOI:** 10.1093/geroni/igae098.3454

**Published:** 2024-12-31

**Authors:** Heather Hunter, Corey L Nagel, Tracie Harrison

**Affiliations:** University of Arkansas for Medical Sciences, Fayetteville, Arkansas, United States; University of Arkansas for Medical Sciences, Little Rock, Arkansas, United States; University of Arkansas for Medical Sciences, Little Rock, Arkansas, United States

## Abstract

The purpose of this study was to examine discrimination reported by middle aged and older non-Hispanic Black Americans by sex, region, type, and frequency throughout the United States. Understanding the contextual and regional influences on recent discriminatory outcomes perceived by older Black adults provides context for future intervention. Hence, we conducted an analysis of 3,761 Non-Hispanic Black adults aged 50+ who participated in the Health and Retirement Study between the years of 2008-2018, examining participants responses to the Perceived Everyday Discrimination Scale. This 6-item scale assesses the frequency of specific types of discrimination with follow up questions that query respondent attributions for experienced discrimination. A binary measure indicating discrimination several times per month or greater was created and used to calculate descriptive statistics stratified by sex and census region of residence. The majority (67%) of respondents reported an experience with one of the listed types of discrimination, with a slightly higher portion of the men (69%) reporting discrimination than the women. Of the sample 28% reported having experienced one of the types of discrimination a few times per month or greater. Highest ranked reasons for discrimination ranged from ancestry (N=200, 29%), gender (N=614, 25%), race (N=1.540, 63%), age (N=998. 41%), financial (N=725, 30%), and disability 465 (19%). Never experiencing discrimination of any type was reported by 34.1% (South), 32.5% (Northeast), 31.9% (Midwest), and 29.8% (West) of respondents by region. Middle aged and older Black Americans perceived discrimination resulting in feelings of less respect, greater fear, heightened threat, and/or poor healthcare.

